# Modeling and Forecasting of Energy Demands for Household Applications

**DOI:** 10.1002/gch2.201900065

**Published:** 2019-11-04

**Authors:** Md. Abdus Salam, Md. Gholam Yazdani, Fushuan Wen, Quazi Mehbubar Rahman, Owais Ahmed Malik, Syeed Hasan

**Affiliations:** ^1^ Department of Electrical and Computer Engineering Faculty of Engineering The University of Western Ontario London N6A 5B9 ON Canada; ^2^ Faculty of Engineering Universiti Teknologi Brunei Bandar Seri Begawan, Tungku Highway Gadong BE1410 Brunei Darussalam; ^3^ Centre for Smart Grid Zhejiang University Hangzhou 310027 China; ^4^ Computer Science Faculty of Science Universiti Brunei Darussalam Bandar Seri Begawan, Tungku Highway Gadong BE1410 Brunei Darussalam

**Keywords:** energy consumption, NAR neural network, number of houses, solar panels, solar water heater, spline and ARIMA models

## Abstract

Energy use is on the rise due to an increase in the number of households and general consumptions. It is important to estimate and forecast the number of houses and the resultant energy consumptions to address the effective and efficient use of energy in future planning. In this paper, the number of houses in Brunei Darussalam is estimated by using Spline interpolation and forecasted by using two methods, namely an autoregressive integrated moving average (ARIMA) model and nonlinear autoregressive (NAR) neural network. The NAR model is more accurate in forecasting the number of houses as compared to the ARIMA model. The energy required for water heating and other appliances is investigated and are found to be 21.74% and 78.26% of the total energy used, respectively. Through analysis, it is demonstrated that 9 m^2^ solar heater and 90 m^2^ of solar panel can meet these energy requirements.

## Graphical abstract

This paper estimates the energy consumptions in residential areas. The study applies Spline interpolation by using two methods: autoregressive integrated moving average (ARIMA) model and nonlinear autoregressive (NAR) neural network. The results show that 21.74% and 78.26% of the total energy are in use for water heating and other applications. The analysis demonstrates that 9 m^2^ solar heater and 90 m^2^ of solar panel can meet these energy requirements.

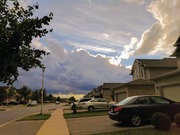

## 1 Introduction

Energy consumption in a building day by day increasing due to household appliances and construction materials. Like any other developed country, Brunei Darussalam is seeing an increase in the population due to its quality liveable conditions. In this regard, the number of housing units needs to be increased to cater for the increased population. This increase in housing units is also increasing to cater for the increased population. This increase in housing units, in turn, is increasing the demand for electrical energy. For any developed country, the residential energy demands at least 36% of the total energy consumption.^1^ In Brunei Darussalam, it is from 49% to 67%, which means a large portion of the generated energy is used in the housing sectors. The saving of energy in this sector will have a positive impact toward the economy of this country. Based on this notion, and the fact that Brunei Darussalam enjoys the high intensity of solar radiation because of its location near the equator. The government of this country has built a solar farm, lately. This firm, called Tenaga Suria Brunei (TSB), in Seria of Kuala Belait district, has a nominal capacity of 1.2 kWh, it covers an area of 12 000 m^2^ with a generation capacity of 1344 MWh. It consists of 9234 pieces of solar panels and supplies power to 200 households. Therefore, the number of solar panels required per house is 46–47. Similar energy saving ideas have been reported in the literature.

A single hidden layer feed‐forward neural network has been used in^2^ to estimate the energy consumption in the building. A building information modeling has been used to predict energy uses in a small‐scale construction in the UK.^3^ Real energy consumption and usages of a room air conditioner in China have been calculated in.^4^

In meeting the energy needs of a selected urban residential building in the three major cities in Egypt, building integrated photovoltaics (BIPV) method have been studied in.^5^ Significant energy savings have been achieved by tracking, monitoring, and detecting abnormal energy consumption behavior of building equipment in.^6^

An automated method for saving energy has been proposed by determining when an electrical device is triggered by households' residents solely from its power trace.^7^ A comparative analysis of energy use in Polish households has been done and compared to selected countries of the European Union in the context of European energy policy.^8^ A method has been proposed to assess the energy performance in four dwellings by tracking and quantifying the effect of zero and low‐cost energy efficiency in Mauritian household.^9^ An efficient graph‐based algorithm for comparison and prediction of household‐level energy use profiles has been presented in.^10^ The performance of a domestic hot water heating system with solar concrete collectors integrated with building structures has been studied and found that the solar water heater with straight and serpentine tubes consumes more area than the other conventional solar water heaters.^11^

The energy performance of a solar water heating system of two different models of storage, namely, fully mixed tank and stratified tank have been studied under Algiers climate conditions and found that the performance of a stratified storage tank is better than the fully mixed tank.^12^ Ahmed Aisa and Tariq Iqbal^13^ have used a solar thermal energy storage system to determine the temperature of a tank and the heat loss of a system for domestic water heating purposes in a detached house setting. A new multiobjective optimization model of combined cooling and heating power (CCHP) system to improve the utilization of renewable energy sources and the shortcoming of solar energy driven by combining solar energy and internal combustion engine has been proposed in.^14^

The long‐term performances of photovoltaic systems have been evaluated with monthly mean weather parameters and hourly solar radiation data using MATLAB program in 10 individual houses situated in the remote site of Ghardaia region.^15^

A novel combined heating system consists of solar kang system and solar air heating system and has been proposed to meet the heating demand in north China and to reduce the pollution of traditional Chinese kang in demonstration buildings that were built in Huzhu, Qinghai province, China.^16^ In this paper, Spline interpolation, ARIMA forecasting models and Nonlinear Autoregressive Neural Networks Time Series Forecast have been used to forecast the number of houses and energy uses.

To address the constant rise in the energy use in Brunei Darussalam, a study on estimating the growing number of houses and the resultant energy consumptions has been presented in this paper. Also, an option of solar energy use to circumvent the energy‐consumption issue has been explored.

## 2 Data Collection

Since the year 1991, in every ten years, the government of Brunei Darussalam has been conducting housing census by types and districts. From the Government of Brunei census book,^17^ the data on the total number of house for the years 1991, 2001, and 2011 for the districts of Brunei‐Muara, Kuala‐Belait, Tutong, and Temburong have been collected for this study, which has been presented in **Table** **1**.

**Table 1:** Number of occupied housing units

Year	Brunei‐Muara	Belait	Tutong	Temburong
1991	24 125	9201	4661	1135
2001	34 775	10 156	6120	1489
2011	45 953	10 559	7241	1640

## 3 Results and Discussion

### 3.1 Spline Interpolation and Time Series Forecasting Models

To estimate the number of houses from 1991 to 2015, two methods namely; Spline Interpolation and ARIMA have been used. The former has been used to interpolate a number of houses for each year from 1991 to 2011, and the latter has been applied to forecast the number of houses from 2012 to 2015.

### 3.2 Spline Interpolation

Spline interpolation is considered as one of the polynomial interpolation methods because the interpolation error can be made smaller even when using low degree polynomials for the spline. In this case, observed data are treated as the “knots or points” by which the spline (i.e., elastic ruler) is bent to pass. The method to mathematically model the shape of the elastic ruler, fixed by (*n* + 1) knots {(*x_i_*,*y_i_*): *i* = 0, 1, ……, *n*} is to interpolate different values between all the pairs of knots (*x_i_*,*y_i_*) and (*x*_*i*+1_, *y*_*i*+1_) with polynomials *y* = *q_i_*(*x*), *i* = 1, 2, …, *n*. These values are calculated by the curvature of a curve *y* = *f*(*x*), which is given by

(1) $$k\;=\;\frac{y^{\prime \prime }}{{\left(1\;+\;{y^{\prime }}^{2}\right)}^{1.5}}$$

where the shape (*k*) of the spline minimizes the bending by passing through all knots where *y*′ and *y*″ will be continuous everywhere at the knots.

### 3.3 ARIMA Time Series Forecast

After using Spline interpolation to estimate missing value from 1991 to 2011, we have a new dataset *X_T_* = (*X*_1_,…, *X_T_*), that can be considered as a time‐series for a length of time *T*, where data each data item is observed periodically by year. In this case, it is necessary to forecast a future value *X*_*T*+*k*_, at (*T* + *k*)th time interval where *k* > 0. An autoregressive integrated moving average (ARIMA) model could be a good method to be applied. For a given time series of data *X_t_*where *t* is an integer index, and the *X_t_* are the real numbers, an autoregressive moving average model, i.e., ARIMA (*p*, *q*) model is given by

(2) $$X_{t}\;-\;\alpha_{1}X_{t-1}\;-\;\cdots \cdot \;-\;\alpha_{p}X_{t-p}\;=\;\varepsilon_{t}\;+\;\theta_{1}\varepsilon_{t-1}\;+\;\cdots \cdot \;+\;\theta_{q}\varepsilon_{t-q}$$

Equation (2) is modified as,

(3) $$\left(1\;-\;\sum_{i=1}^{p}\alpha_{i}L^{i}\right)X_{t}\;=\;\left(1\;+\;\sum_{i=1}^{q}\theta_{i}L^{i}\right)\varepsilon_{t}$$

where *L* is the lag operator (i.e. *LX_t_* = *X*_*t*−1_ for all *t* > 1), α_*i*_ are the parameters of the autoregressive part of the model, θ_*i*_ are the parameters of the moving average part and ε_*t*_ are the error terms. The error terms ε_*t*_ are generally assumed to be independent, and identically distributed random variables sampled from a normal distribution with zero means. Assuming that the polynomial has a unit root (a factor of (1 − *L*)) of multiplicity *d*. Equation (3) can be modified as

(4) $$\left(1\;-\;\sum_{i=1}^{p}\alpha_{i}L^{i}\right)X_{t}\;=\;\left(1\;+\;\sum_{i=1}^{p-d}\phi_{i}L^{i}\right){\left(1\;-\;L\right)}^{d}$$

Then ARIMA (*p*, *d*, *q*) is defined as

(5) $$\left(1\;-\;\sum_{i=1}^{p}\phi_{i}L^{i}\right){\left(1\;-\;L\right)}^{d}X_{t}\;=\;\delta \;+\;\left(1\;+\;\sum_{i=1}^{q}\theta_{i}L^{i}\right)\varepsilon_{t}$$

Let M be a matrix that contains data represented in Table 1, where M's column is Table 1's column.

For each column from “Muara” to “Temburong,” the SPLINE model is used to interpolate data from 1991 to 2011 (interval *n* = 21) with three given values on 1991, 2001, and 2011. The result is tabulated in **Table** **2**.

**Table 2:** SPLINE interpolation

Year	Muara	Belait	Tutong	Temburong
1991	24 125	9201	4661	1135
1992	25 166.24	9321.34	4822.11	1179.535
1993	26 212.76	9436.16	4979.84	1222.04
1994	27 264.56	9545.46	5134.19	1262.515
1995	28 321.64	9649.24	5285.16	1300.96
1996	29 384	9747.5	5432.75	1337.375
1997	30 451.64	9840.24	5576.96	1371.76
1998	31 524.56	9927.46	5717.79	1404.115
1999	32 602.76	10 009.16	5855.24	1434.44
2000	33 686.24	10 085.34	5989.31	1462.735
2001	34 775	10 156	6120	1489
2002	35 869.04	10 221.14	6247.31	1513.235
2003	36 968.36	10 280.76	6371.24	1535.44
2004	38 072.96	10 334.86	6491.79	1555.615
2005	39 182.84	10 383.44	6608.96	1573.76
2006	40 298	10 426.5	6722.75	1589.875
2007	41 418.44	10 464.04	6833.16	1603.96
2008	42 544.16	10 496.06	6940.19	1616.015
2009	43 675.16	10 522.56	7043.84	1626.04
2010	44 811.44	10 543.54	7144.11	1634.035
2011	45 953	10 559	7241	1640

In Table 2, each district column is considered as a time‐series to predict its value from the year 2012 to 2015, using ARIMA (0,0,0) model (i.e., white noise model).

**Tables** **3** **–** **6** forecast number of the house from the year 2012 to 2015 for Muara, Tutong, Kuala‐Belait, and Temburong districts, respectively.

**Table 3:** Brunei‐Muara forecast number of houses

Year	Forecast	Lo_80	Hi_80	Lo_95	Hi_95
2012	47 094.56	47 082.99	47 106.13	47 076.87	47 112.25
2013	48 236.12	48 210.25	48 261.99	48 196.56	48 275.68
2014	49 377.68	49 334.39	49 420.97	49 311.48	49 443.88
2015	50 519.24	50 455.87	50 582.61	50 422.33	50 616.15

**Table 4:** Tutong forecast number of houses

Year	Forecast	Lo_80	Hi_80	Lo_95	Hi_95
2012	7337.89	7333.185	7342.595	7330.694	7345.086
2013	7434.78	7424.258	7445.302	7418.689	7450.871
2014	7531.67	7514.064	7549.276	7504.744	7558.596
2015	7628.56	7602.788	7654.332	7589.144	7667.976

**Table 5:** Kuala‐Belait forecast number of houses

Year	Forecast	Lo_80	Hi_80	Lo_95	Hi_95
2012	10 574.46	10 566.45	10 582.47	10 562.21	10 586.71
2013	10 589.92	10 572.02	10 607.82	10 562.54	10 617.3
2014	10 605.38	10 575.42	10 635.34	10 559.56	10 651.2
2015	10 620.84	10 576.98	10 664.7	10 553.77	10 687.91

**Table 6:** Temburong forecast number of houses

Year	Forecast	Lo_80	Hi_80	Lo_95	Hi_95
2012	1645.965	1643.326	1648.604	1641.929	1650.001
2013	1651.93	1646.029	1657.831	1642.905	1660.955
2014	1657.895	1648.02	1667.77	1642.792	1672.998
2015	1663.86	1649.404	1678.316	1641.752	1685.968

**Figure 1:**
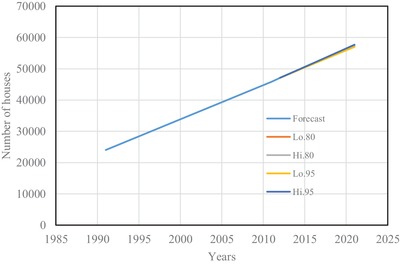
Number of houses for Muara district.

**Figure 2:**
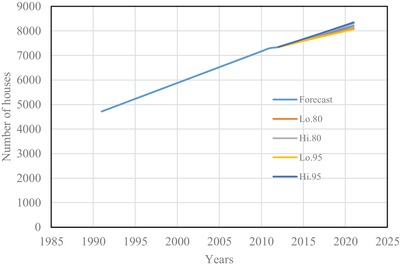
Number of houses for Tutong district.

**Figure 3:**
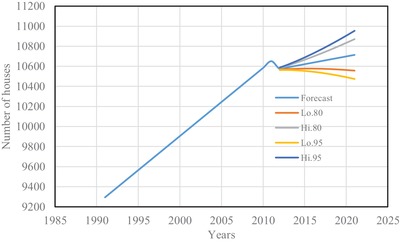
Number of houses for Kuala‐Belait district.

**Figure 4:**
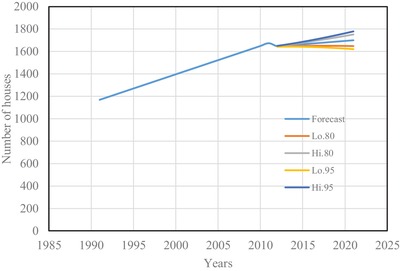
Number of houses for Temburong district.

The estimated number of houses using Spline interpolation and ARIMA model are shown from **Figures** **1** to **4** for four districts in Brunei Darussalam. From Figures 1 to 4, it is observed that the increased number of houses for four districts is linear with different rate of increase for the census period of 1991 to 2011. The forecasting number of houses is more agreeable with the census data. The estimated number of houses from the two models is tabulated in **Table** **7**. Using these data, the following computations have been carried out. The electrical energy generation and consumption data for different districts of Brunei Darussalam have been collected from Brunei Darussalam Statistical Year Books 2010, 2014, and 2015, as presented in **Table** **8**, while **Table** **9** presents water production and consumption data, collected from the same year books.

**Table 7:** Estimated number of houses

Year	Brunei‐Muara	Tutong	Kuala Belait	Temburong	Total
2005	39 182.84	6608.96	10 383.44	1573.76	57 749
2006	40 298	6722.75	10 426.5	1589.875	59 037.13
2007	41 418.44	6833.16	10 464.04	1603.96	60 319.6
2008	42 544.16	6940.19	10 496.06	1616.015	61 596.43
2009	43 675.16	7043.84	10 522.56	1626.04	62 867.6
2010	44 811.44	7144.11	10 543.54	1634.035	64 133.13
2011	45 953	7241	10 559	1640	65 393
2012	47 094.56	7337.89	10 574.46	1645.965	66 652.88
2013	48 236.12	7434.78	10 589.92	1651.93	67 912.75
2014	49 377.68	7531.67	10 605.38	1657.895	69 172.63
2015	50 519.24	7628.56	10 620.84	1663.86	70 432.5

**Table 8:** Energy generation and consumption

Year	Total houses	Energy generation [GWh]	Energy consumption [GWh]	Residential energy use [%]	Per house consumption [MWh]
2005	57 797	2912.8	1960.3	67.3	33.9
2006	59 077	3298.3	1649.7	50.0	27.9
2007	60 348	3394.8	1768.0	52.1	29.3
2008	61 613	3423.5	1870.1	54.6	30.4
2009	62 870	3611.5	1955.0	54.1	31.1
2010	64 120	3792.2	1887.2	49.8	29.4
2011	65 361	3723.0	1935.6	52.0	29.6
2012	66 595	3928.7	1997.9	50.9	30.0
2013	67 822	3961.8	1986.1	50.1	29.3
2014	69 041	4054.6	2106.4	52.0	30.5
2015	70 254	4198.8	2231.3	53.1	31.8

**Table 9:** Water production and consumption

Year	Total houses	Water production [Thousand m^3^]	Water consumption [Thousand m^3^]	House hold consumption [%]	Per house consumption [m^3^]
2006	59 077	138 263	47 892	34.6	810.7
2007	60 348	137 712	51 478	37.4	853.0
2008	61 613	142 475	54 783	38.5	889.1
2009	62 870	151 514	63 227	41.7	1005.7
2010	64 120	160 234	68 262	42.6	1064.6
2011	65 361	166 161	66 781	40.2	1021.7
2012	66 595	167 396	92 789	55.4	1393.3
2013	67 822	162 990	80 819	49.6	1191.6
2014	69 041	166 129	90 343	54.4	1308.5
2015	70 254	152 471	87 719	57.5	1248.6

### 3.4 Nonlinear Autoregressive Neural Networks Time Series Forecast

A nonlinear autoregressive (NAR) neural network is a type of dynamic neural networks which includes the tapped delay lines used for nonlinear forecasting.^18^, ^19^, ^20^ A NAR neural network predicts a time series *y* at time *t* (*y(t)*), given *d* past values of *y(t)* as follows

(6) y(t) = f(y(t − 1) + y(t − 2) + −−− + y(t − d)) + ε(t)

**Figure 5:**
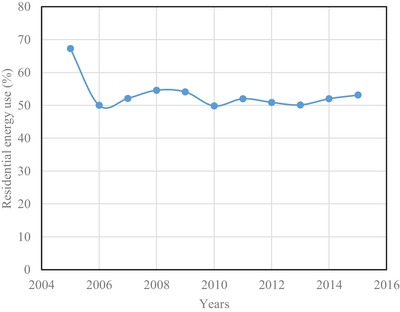
Architecture of nonlinear autoregressive neural network.

The function *y*(..)is approximated by training of the neural network by optimizing the network weights and neuron bias and ε(*t*) is the error of the approximation of the series *y* at time *t*. **Figure** **5** shows an architecture of NAR neural network for forecasting *y*(*t*) based on *d* = 4 with one hidden layer consisting of 10 neurons and one output layer neuron. The decision about the number of hidden layers and neurons per layer depends on the training data and can be optimized through various configurations (i.e., different number of hidden layers and number of neuron in each layer) to minimize the prediction error. In this paper, the NAR neural network was implemented using Neural Network Toolbox in MATLAB R2016a. The network was created and trained in open loop form with one hidden layer (consisting of 10 neurons), and different values for the delay were tested. Training with open loop prediction is more efficient than with closed loop prediction due to the flexibility of providing the network with correct feedback inputs in order to produce the correct feedback outputs. After the training phase, the network was converted to closed loop form for predicting the number of houses for the next four years.

Three learning algorithms, namely Levenberg–Marquardt, Bayesian Regularization and Scaled Conjugate Gradient were applied for training, and their results were compared. Levenberg–Marquardt is a Jacobian calculation based backpropagation procedure which is time efficient but it requires more memory space. The training phase automatically stops when an increase in the mean squared error of the validation samples is detected. Bayesian Regularization is also a Jacobian calculations based algorithm which provides better results for difficult, noisy and small datasets. However, this algorithm typically requires more time for training. The training phase for this algorithm is stopped according to the adaptive weight minimization (regularization) criterion. Scaled Conjugate Gradient is suitable for large problems because it uses gradient calculations, which requires less memory. Hence, this algorithm is more memory efficient as compared to previous techniques. The training for this algorithm automatically stops when an increase in the mean squared error of the validation samples is detected.

In this study, NAR neural networks were used to build a time series model for estimating and forecasting the number of houses in different districts of Brunei Darussalam. The network structure contains one input (i.e., number of houses at time *t* − 1), and one output (i.e., the number of houses for time *t*). The number of delays was determined based on the values of mean squared error (the average squared difference between outputs and targets) and the regression value (it measures the correlation between outputs and targets) after training and testing phases.

**Figure 6:**
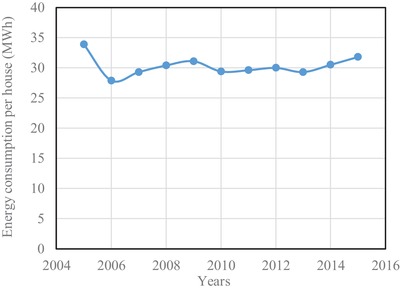
A sample mean squared error results (training, validation, and testing) of Levenberg–Marquardt based NAR neural network.

**Figure 7:**
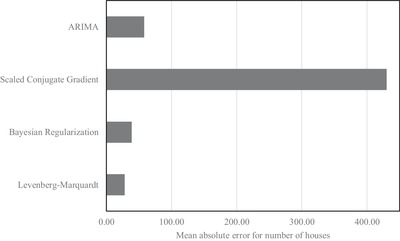
Comparison of mean absolute error (number of houses) for NAR and ARIMA models.

**Figure 8:**
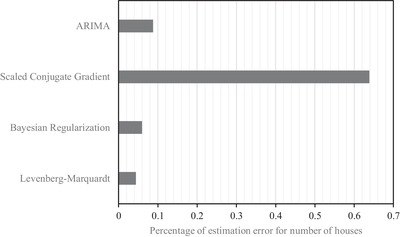
Comparison of mean absolute value of percentage of estimation error for number of houses for NAR and ARIMA models.

Three NAR neural networks were implemented by dividing the data randomly into training (70%), validation (15%), and testing (15%) groups. Different values of delays were tested, and based on the minimum value of mean squared error and maximum value of regression coefficient, the delay was set to 4 for prediction of a number of houses. An example of training, validation and testing performance curves (mean squared error values) for the Levenberg–Marquardt NAR model is shown in **Figure** **6**. **Tables** **10** to **13** show the forecast of a number of houses for four districts of Brunei Darussalam for the years 2012 to 2015. **Tables** **14** to **16** show the total number of estimated houses for the year 2005 to 2015 using three different training algorithms (Levenberg–Marquardt, Bayesian Regularization, and Scaled Conjugate Gradient). Moreover, the estimation error for a number of houses and percentage of estimation error for a number of houses have been shown in **Tables** **17** and **18**, respectively, for three NAR neural networks and ARIMA model. **Figures** **7** and **8** indicate that the lowest value of mean absolute error for estimation of a number of houses was found for Levenberg–Marquardt NAR model while the highest value of mean absolute error for estimation of the number of houses was observed for Scaled Conjugate Gradient NAR model. These figures also suggest that the nonlinear models (Levenberg–Marquardt, Bayesian Regularization) are more accurate in estimating the number of houses as compared to the linear ARIMA model.

**Table 10:** Brunei‐Muara forecast number of houses using NAR

Year	Levenberg–Marquardt	Bayesian Regularization	Scaled Conjugate Gradient
2012	47 078.82	47 093.4	46 676.6
2013	48 187.57	48 237.82	47 440.22
2014	49 291.29	49 383.52	47 802.65
2015	50 358.59	50 529.66	48 776.92

**Table 11:** Tutong forecast number of houses using NAR

Year	Levenberg–Marquardt	Bayesian Regularization	Scaled Conjugate Gradient
2012	10 569.59	10 568.93	10 567.53
2013	10 574.64	10 573.31	10 572.97
2014	10 575.89	10 572.12	10 579.03
2015	10 571.80	10 565.28	10 584.61

**Table 12:** Kuala‐Belait forecast number of houses using NAR

Year	Levenberg–Marquardt	Bayesian Regularization	Scaled Conjugate Gradient
2012	7334.58	7334.64	7397.34
2013	7425.18	7424.98	7566.58
2014	7513.66	7512.07	7660.44
2015	7601.57	7595.96	7812.39

**Table 13:** Temburong forecast number of houses using NAR

Year	Levenberg–Marquardt	Bayesian Regularization	Scaled Conjugate Gradient
2012	1643.93	1643.93	1643.11
2013	1645.81	1645.82	1644.11
2014	1645.56	1645.66	1643.72
2015	1642.99	1643.43	1642.65

**Table 14:** Estimated number of houses using Levenberg–Marquardt

Year	Brunei‐Muara	Tutong	Kuala Belait	Temburong	Total
2005	39 182.84	6608.96	10 383.5	1573.76	57 749.06
2006	40 298	6722.75	10 426.55	1589.87	59 037.17
2007	41 418.44	6833.16	10 464.08	1603.96	60 319.64
2008	42 544.18	6940.19	10 496.09	1616.01	61 596.47
2009	43 675.16	7043.84	10 522.57	1626.04	62 867.61
2010	44 810.56	7144.11	10 543.59	1634.03	64 132.29
2011	45 947.29	7241	10 559.22	1640	65 387.51
2012	47 078.82	7334.58	10 569.59	1643.93	66 626.92
2013	48 187.57	7425.18	10 574.64	1645.81	67 833.2
2014	49 291.29	7513.66	10 575.89	1645.56	69 026.4
2015	50 358.59	7601.57	10 571.8	1642.99	70 174.95

**Table 15:** Estimated number of houses using Bayesian Regularization

Year	Brunei‐Muara	Tutong	Kuala Belait	Temburong	Total
2005	39 184.44	6608.97	10 383.44	1573.76	57 750.61
2006	40 300.14	6722.75	10 426.5	1589.88	59 039.27
2007	41 420.77	6833.14	10 464.04	1603.96	60 321.91
2008	42 546.2	6940.16	10 496.06	1616.01	61 598.43
2009	43 676.32	7043.81	10 522.56	1626.04	62 868.73
2010	44 810.98	7144.1	10 543.54	1634.04	64 132.66
2011	45 950.06	7241.04	10 559	1640	65 390.1
2012	47 093.4	7334.64	10 568.93	1643.93	66 640.9
2013	48 237.82	7424.98	10 573.31	1645.82	67 881.93
2014	49 383.52	7512.07	10 572.12	1645.66	69 113.37
2015	50 529.66	7595.96	10 565.28	1643.43	70 334.33

**Table 16:** Estimated number of houses using Scaled Conjugate Gradient

Year	Brunei‐Muara	Tutong	Kuala Belait	Temburong	Total
2005	39 071.7	6610.4	10 397.48	1566.13	57 645.71
2006	40 264.24	6723.94	10 438.69	1586.57	59 013.44
2007	41 526.61	6828.15	10 471.58	1604.34	60 430.68
2008	42 791.46	6923.75	10 499.18	1618.3	61 832.69
2009	43 978.18	7019.98	10 522.61	1628.51	63 149.28
2010	45 028.81	7129.32	10 541.88	1635.6	64 335.61
2011	45 924.16	7256.91	10 556.82	1640.27	65 378.16
2012	46 676.6	7397.34	10 567.53	1643.11	66 284.58
2013	47 440.22	7566.58	10 572.97	1644.11	67 223.88
2014	47 802.65	7660.44	10 579.03	1643.72	67 685.84
2015	48 776.92	7812.39	10 584.61	1642.65	68 816.57

**Table 17:** Estimation error for number of houses

Year	Levenberg–Marquardt	Bayesian Regularization	Scaled Conjugate Gradient	ARIMA
2005	−47.94	−46.39	−151.29	−48.00
2006	−39.83	−37.73	−63.56	−39.87
2007	−28.36	−26.09	82.68	−28.40
2008	−16.53	−14.57	219.69	−16.57
2009	−2.39	−1.27	279.28	−2.40
2010	12.29	12.66	215.61	13.13
2011	26.51	29.10	17.16	32.00
2012	31.92	45.90	−310.42	57.88
2013	11.20	59.93	−598.12	90.75
2014	−14.60	72.37	−1355.16	131.63
2015	−79.05	80.33	−1437.43	178.50
MAE	28.24	38.76	430.04	58.10

**Table 18:** Percentage of estimation error for number of houses

Year	Levenberg–Marquardt	Bayesian Regularization	Scaled Conjugate Gradient	ARIMA
2005	−0.0829	−0.0803	−0.2618	−0.0830
2006	−0.0674	−0.0639	−0.1076	−0.0675
2007	−0.0470	−0.0432	0.1370	−0.0471
2008	−0.0268	−0.0236	0.3566	−0.0269
2009	−0.0038	−0.0020	0.4442	−0.0038
2010	0.0192	0.0197	0.3363	0.0205
2011	0.0406	0.0445	0.0263	0.0490
2012	0.0479	0.0689	−0.4661	0.0869
2013	0.0165	0.0884	−0.8819	0.1338
2014	−0.0211	0.1048	−1.9628	0.1907
2015	−0.1125	0.1143	−2.0460	0.2541

### 3.5 Household Energy Consumption

**Figure 9:**
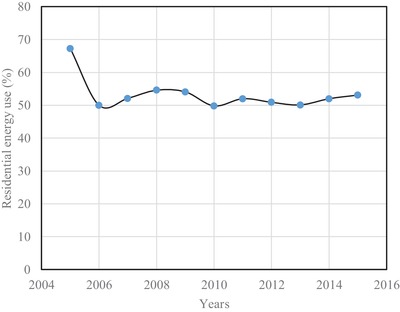
Variation of residential energy use.

**Figure 10:**
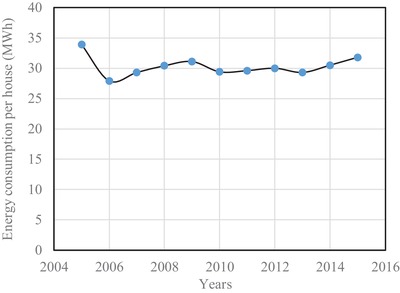
Energy consumption per house.

In Brunei Darussalam, the major energy consuming sectors are the residential and commercial ones. The percentage of energy consumption in the residential sector from the year 2005 to 2015 is shown in **Figure** **9**. It is seen that the percentage of energy consumption in the year 2005 is 67.3. Apart from this, energy consumption has been found to be hovering around 50% up to the investigated the year 2015. This means that a major portion of electrical energy is consumed in the residential sector alone. The energy consumption per house is shown in **Figure** **10**. The energy consumption per house in the year 2005 was at its highest, which was 33.9 MWh. Whereas the energy consumption from the year 2006 to 2015 has been found to 30 MWh on average.^17^ Though the energy between 2006 and 2015 has been found to be almost steady, the total consumption sees an increase since the number of houses increases at a rate of 1.94% from year to year.

### 3.6 Household Water Consumption

**Figure 11:**
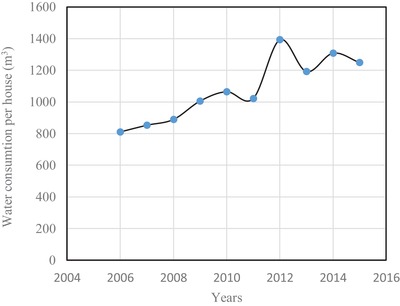
Water consumption per house hold.

**Figure 12:**
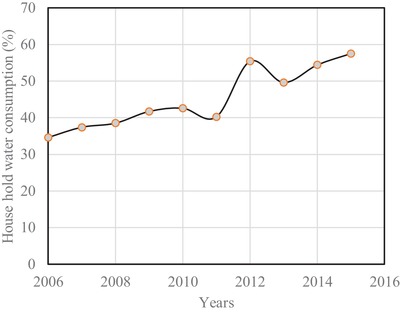
Percentage of water consumption per house hold.

**Figure 13:**
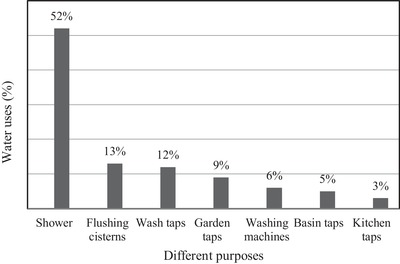
Specific purposes water consumption.^21^

Annual water consumptions in m^3^ per house are plotted in **Figure** **11**. This annual water consumption per house shows an increasing trend starting from 810.7 to 1064 m^3^ from the year 2006 to 2010.^17^ Afterward, there is an abrupt increase from 2011 to 2012 due to weather condition. It then decreases and remains at an average value of 1250 m^3^. The overall yearly rate of increase in water consumption has been calculated and found to be 6% per household. The water consumption is divided into two categories, namely, house hold and commercial. The percentage of water use per house is shown in **Figure** **12**. Here, it observed that the household percentage of water consumption has an increasing rate starting at 34.6% to 57.5%. However, there is a sharp increase from 40.2% to 55.4% for the year 2006 to 2012. From Figures 7 and 8, it can be anticipated that the demand for domestic energy to warm up the water will increase in future. On average, the water consumption in a house for different water‐related purposes is shown in **Figure** **13**. It is seen that 52% of total water consumption per house results from everyday shower‐use.

Generally, warm water is preferred in the shower, and this requires electrical energy overhead. In this case, the amount of required electrical energy needs to be calculated by using Equation (1). The volume of the water to be heated in m^3^ is calculated from the water consumption in m^3^ multiplied by the percentage of water use in the shower (52%) using Figures 11 and 13, respectively. It is then converted to mass (*m*) in kg by multiplying the calculated volume with the standard water density. For each year of investigation, the electrical energy required to warm up the water *W*_HE_ is calculated as

(7) $$W_{\mathrm{HE}}=\;2.77\;\times \;10^{-4}m\;C_{p}\;\Delta T$$

where *W*_HE_ in kWh, *m* is the mass of water in kg, *C*_p_ is the specific heat of the water in kJ kg^−1^ K^−1^, Δ*T* is the temperature difference between the supply‐water and the room temperature.

The percentage of energy use for heating the water is calculated from the ratio between *W*_HE_, and the total consumed energy used from Figure 10.

**Figure 14:**
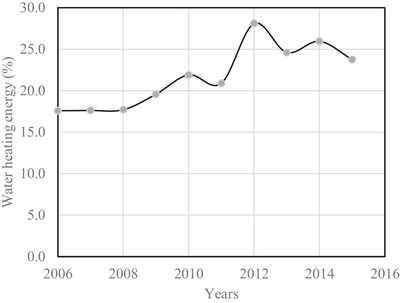
Percentage water consumption per house hold.

The percentage of electrical energy required to heat the water for every house from the year 2006 to 2015 have been plotted in **Figure** **14**.^20^ The highest percentage of electrical energy required to heat the water is found to be 28% in the year 2012.

**Figure 15:**
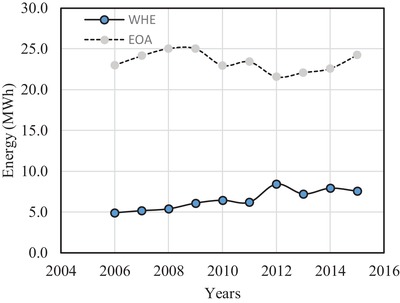
Energy for different purposes.

The energy consumption in the other appliances (EOA) in a house is calculated by subtracting the energy used for water‐heating from the total consumed energy per house. The energy for heating water and other appliances are plotted together in **Figure** **15**.

### 3.7 Solar Energy as an Option to Cut Down the Energy Expenses

Solar energy can be used in cutting down the cost associated with energy. It can be used in two ways, namely, direct heating and electricity generation by using a solar panel. In Brunei, the roof area of a typical two‐storied house is around 500 m^2^. Part of this roof area will be used for solar heating and solar power generation. The collector yield *C*_y_ is calculated as

(8) $$C_{y}\;=\;S_{R}\;\times \;\eta_{c}\;\times \;\eta_{\mathrm{sys}}$$

where *C*_y_ is the collector yield, kWh day^−1^ m^−2^, *S*_R_ is the solar radiation per day m^2^, η_c_ is the efficiency of the collector, η_sys_ is the efficiency of the system.

Averaging the solar radiation data from 2010 to 2011, solar radiation is found to be 4.96 kWh day^−1^ m^−2^. Assuming the collector and the system efficiencies 0.61 and 0.85, respectively. The collector yield is found to be 2.57 kWh day^−1^ m^−2^. The energy demand per day (*Q*_d_) can be determined as

(9) $$Q_{d}=\;\frac{W_{\mathrm{HE}}\;\times \;1000}{365}$$

By considering the highest water heating value, per day energy has been found to be 23 kWh. The required area of the solar collector (*A*_c_) can be determined as

(10) $$A_{c}\;=\;\frac{Q_{d}}{C_{y}}$$

The area of the collector has been found to be 8.95 m^2^. The rest of the area can be used for the solar panel to generate electricity for other appliances at the house.

Polycrystalline Silicon with a dimension of 1.956 m × 0.992 m can generate a maximum solar power of 300 W and a resultant current of 15 A. Using the minimum sunshine hour of 5 h, the energy generation per day (*E*_G_) can be calculated as

(11) $$E_{G}=\;\frac{300\;\times \;5}{1000}\;=\;1.5\;\mathrm{kWh}$$

The per day energy demand (*E*_DS_) from the solar panel can be determined as

(12) $$E_{\mathrm{DS}}=\;\frac{\mathrm{EOA}\;\times \;1000}{365}$$

For a maximum energy value of 25 MWh per year energy of the other appliances, the per day energy demand from the solar panel is found to be 68.5 kWh. Therefore, the number of the required solar panel (*N*_SP_)is determined as

(13) $$N_{\mathrm{SP}}=\;\frac{E_{\mathrm{DS}}}{E_{G}}$$

In this case, the number solar panels are to be 46 with an area of 89.24 m^2^ which is in an agreement with an established solar energy plant named as “Tenaga Suria Brunei (TSB)” located in Seria Kuala Belait district.

The plant at TSB covers an area of 12 000 m^2^ with a total number of 9234 solar panels supplying 200 households with a generation of 1344 MWh per year. It has a rated capacity of 1.2 MWp. The yearly yield factor (*Y*_f_) is calculated as

(14) $$Y_{f}\;=\;\frac{{\mathrm{EG}}_{a}}{P_{r}}\;=\;\frac{1344}{1.2}\;=\;1120\;h$$

where EG_a_ is the generation per year in MWh, *P*_r_ is the rated power in MW.

## 4 Conclusion

The number of houses in the four districts of Brunei Darussalam is estimated from the census data using two models. The mean absolute error for the number of houses have been determined using three different training algorithms, namely, Levenberg–Marquardt, Bayesian Regularization and Scaled Conjugate Gradient and ARIMA model. The values of mean absolute errors for those methods are found to be 0.0442, 0.0594, 0.6388, and 0.0876, respectively. In addition, the energy consumption per household is found out from the energy consumption data and the estimated number of houses. The average consumption per household is found to be 30.3 MWh for the investigated year from 2005 to 2015. This amount of electrical power is proposed to generate using solar panels, which has an annual yield factor of 1120 h. This consumption is divided into two broad categories, namely, water heating and other appliances. The energy required for water heating purposes is 21.74%, whereas the energy required for other appliances is 78.26%. It has been found that the solar water heater of 9 m^2^, and the solar panel of 90 m^2^ are required to meet the necessary energy demand for any household with the rooftop area of 500 m^2^.

## Conflict of Interest

The authors declare no conflict of interest.

## References

[1] Tenaga Suria Brunei , 1.2 MW Brunei Solar Power, 26 May 2011.

[2] S. Naji , A. Keivani , S. Shamshirband , U. J. Alengaram , M. Z. Jumaat , Z. Mansor , M. Lee , Energy 2016, 97, 506.

[3] F. H. Abanda , L. Byers , Energy 2016, 97, 517.

[4] J. Wu , C. Liu , H. Li , D. Ouyang , J. Cheng , Y. Wang , S. You , Energy 2017, 119, 1036.

[5] M. Kares , P. Singh , IEEE Global Humanitarian Tech. Conf., Seattle, USA, 2016, pp. 413–420.

[6] Z. Li , Y. Han , P. Xu , Appl. Energy 2014, 124, 325.

[7] H.‐Â. Cao , T. K. Wijaya , K. Aberer , N. Nunes , IEEE Int. Conf. on Big Data, Washington, USA, 2016, pp. 1301–1308.

[8] M. Kott , IEEE Modern Electric Power Systems, Wroclaw, Poland, 2015, pp. 1–5.

[9] Y. K. Ramgolam , A. Needroo , IEEE AFRICON, Pointe‐Aux‐Piments, Mauritius, 2013, pp. 1–5.

[10] N. Charlton , D. V. Greetham , C. Singleton , IEEE Int. Workshop on Intelligent Energy Systems, Vienna, Austria, 2013, pp. 119–124.

[11] M. Edwin , U. Arunachalam , R. Rakesh , M. Sanjeev Srinivas , A. Santhana Kumar , Int. Conf. on Energy Efficient Technology for Sustainability, Nagercoil, India, 2016, pp. 223–227.

[12] M. Missoum , A. Hamidat , K. Imessad , S. Bensalem , A. Khodja , IEEE 7th Int. Energy Congress, Hammamet, Tunisia, 2016, pp. 1–6.

[13] A. Aisa , T. Iqbal , IEEE 7th Annual Information Technology, Electronics and Mobile Communication Conf., Vancouver, Canada, 2016, pp. 1–9.

[14] Z. Tao , S. Tingting , L. Xin , P. Wangyang , IEEE 11th Conf. of Industrial Electronics and Applications, Hefei, China, 2016, pp. 1127–1130.

[15] M. Koussa , D. Saheb , S. Hadji , Int. Renewable and Sustainable Energy Conf., Ouarzazate, Morocco, 2014, pp. 253–257.

[16] D. Zhaoa , J. Jia , H. Yub , W. Weia , H. Zheng , Energy Build. 2017, 143, 61.

[17] Members , Department of Economic Planning and Development, Brunei Darussalam, 2016.

[18] L. R. Medsker , L. C. Jain , Recurrent Neural Networks: Design and Applications, CRC Press, Boca Raton, FL 2000.

[19] Q. Cao , B. T. Ewing , M. A. Thompson , Eur. J. Oper. Res. 2012, 221, 148.

[20] M. Ibrahim , S. Jemei , G. Wimmer , D. Hissel , Electr. Power Syst. Res. 2016, 136, 262.

[21] Z. A. Hishamuddin , P. Z. Ibrahim , Conf. of ASEAN Federation of Engineering Organizations (CAFEO 31), Jakarta, Indonesia, 2013.

